# Orally Derived Stem Cell-Based Therapy in Periodontal Regeneration: A Systematic Review and Meta-Analysis of Randomized Clinical Studies

**DOI:** 10.3390/dj12050145

**Published:** 2024-05-16

**Authors:** Alessandro Campagna, Giacomo Baima, Federica Romano, Federico Amoroso, Federico Mussano, Giacomo Oteri, Mario Aimetti, Matteo Peditto

**Affiliations:** 1 Department of Biomedical and Dental Sciences and Morphofunctional Imaging, University of Messina, 98122 Messina, Italy; alexcampagna@hotmail.it (A.C.); giacomo.oteri@polime.it (G.O.); matteo.peditto@unime.it (M.P.); 2 Department of Surgical Sciences, C.I.R. Dental School, University of Turin, 10126 Torino, Italy; giacomo.baima@unito.it (G.B.); federica.romano@unito.it (F.R.); federico.amoroso@unito.it (F.A.); mario.aimetti@unito.it (M.A.); 3 Politecnico di Torino, 10129 Torino, Italy

**Keywords:** periodontitis, periodontal regeneration, stem cells, biomaterials

## Abstract

The present systematic review was performed to assess the application of orally derived stem cells in periodontal regenerative therapy, and because of this, the following PICO question was proposed: “In patients with periodontitis, can the adjunctive use of orally derived stem cells provide additional clinical and radiographic benefits for periodontal regeneration?”. Randomized clinical studies were electronically and manually searched up until December 2023. Quantitative analyses were performed with the aim of evaluating the mean differences (MDs) between the treatment and control groups in terms of clinical attachment level (CAL) gain, probing pocket depth (PPD) reduction, gingival recession (GR), and radiographic bone gain (RBG) using random effect models. A total of seven studies were selected for the systematic review. Meta-analyses excluding studies with a high risk of bias highlighted a non-statistically significant result for the use of stem cells when compared to the control groups in terms of CAL gain [MD = 1.05; 95% CI (−0.88, 2.97) *p* = 0.29] and PPD reduction [MD = 1.32; 95% CI (−0.25, 2.88) *p* = 0.10]. The same also applied to GR [MD = −0.08; 95% CI (−0.79, 0.63) *p* = 0.83] and RBG [MD = 0.50; 95% CI (−0.88, 1.88) *p* = 0.48]. Based on the high heterogeneity, there is not enough evidence to consider the adjunctive application of orally derived mesenchymal stem cells as a preferential approach for periodontal regenerative treatment, as compared to standard procedures.

## 1. Introduction

Periodontitis is a biofilm-mediated disease with an intrinsic inflammatory component, which causes the progressive breakdown of the supporting periodontal tissues [1,2,3]. The objectives of the first steps of periodontal therapy are the control of the microbial infection and the resolution of the inflammation, which clinically refers to the absence of bleeding on probing (BoP) and the presence of shallow probing pocket depths (PPD ≤ 4 mm) [4,5,6]. However, residual pockets often persist after non-surgical treatment, especially in sites with furcation involvement (FI) and/or deep intrabony defects [7,8]. After the XVI European Workshop in Periodontology, there is a strong recommendation to treat dental elements with deep residual PPD associated with intrabony defects of ≥3 mm via the use of periodontal regenerative surgery [9,10,11]. Similarly, the recommendation was expressed for the treatment of class II maxillary and mandibular molars [9]. As reported in the literature, the various surgical techniques and biomaterials developed in the last 30–40 years with the aim of predictable periodontal regeneration have achieved variable success [12,13,14]. The benefits reported are often limited to deep intrabony defects and class II mandibular FI, while supracrestal defects, non-containing intrabony defects, and maxillary class II or III FI still have less predictable outcomes [15,16,17,18]. For this reason, new tissue engineering strategies are being sought, and the implementation of innovative techniques using orally derived stem cells is growing in terms of scientific research in periodontology [19,20].

When compared to biomaterials, which are scaffolds characterized by unique chemical, mechanical, and biological properties, mainly osteoinductivity and osteoconductivity [21], cell therapy relies on replenishing and/or empowering the inner healing body potential [22,23]. In recent years, cell regeneration therapy has been introduced in many areas of medicine, such as cardiology, neurology, or traumatology [24,25], as well as for the treatment of orofacial dystrophies, diabetic problems, and autoimmune diseases [26,27]. Regenerative medicine commonly employs stem cells, particularly mesenchymal stem cells (MSCs), which possess unique faculties, like self-renewal, clonality, and potency. These adult stem cells exhibit anti-inflammatory properties and contribute to tissue repair processes, secreting mediators with various beneficial effects [28,29]. The expression of specific surface antigens, including CD44, CD73, CD90, and CD105, helps characterize MSCs, while lacking hematopoietic and endothelial markers [30]. Indeed, MSCs are defined by their plastic adherence, capacity of self-renovation, and the potential for differentiation in vitro into different types of cells, like osteoblasts, adipocytes, and chondroblasts, under specific stimuli [31,32]. Many intraoral and dental sources of MSCs are available, for example, dental pulp, periodontal ligament, bone marrow from alveolar bone, dental follicle, gingival connective tissue, or apical papilla [33,34,35,36]. In virtue of their self-renewal, multipotentiality, immunomodulation, and tissue regeneration capacities, MSCs can promote the growth of various periodontal tissues, like alveolar bones, root cementum, and periodontal ligaments, even in situations with low intrinsic potential [23,37,38,39,40,41]. A recent study assessed periodontal regenerative approaches in animal models, observing that mesenchymal stem cells used alone or mixed with other biomaterials, such as bovine bone, beta-tricalcium phosphate, or platelet-rich plasma, offered better regenerative outcomes than those of the group with biomaterials alone [42]. Most preclinical studies have indeed supported the biological rationale of employing MSCs to promote osteoinduction and tenogenesis, while simultaneously decreasing inflammation [26,43,44,45].

In humans, recent systematic reviews evaluated the clinical results of periodontal regeneration with MSCs derived from different sources [46,47], reporting a significant advantage of using cell therapy in terms of the final outcomes. However, due to the presence of highly heterogeneous results and the detection of methodological inconsistencies in data handling, the purpose of the present systematic review was to elucidate the adjunctive clinical and radiographic effects of using orally derived stem cells for periodontal regeneration through a meta-analytic approach.

## 2. Materials and Methods

A systematic review protocol was written in the planning stages, and was registered on the international prospective register of systematic reviews (PROSPERO; CRD42024525702). The PRISMA statement was followed in both the planning and reporting of the review [48].

### 2.1. Focused Question

This systematic review aimed to answer the following PICO question: “In patients with periodontitis, can the adjunctive use of orally derived stem cells provide additional clinical benefits measured as clinical attachment level (CAL) gain, probing pocket depth (PPD) reduction, recession (GR) and radiographic bone gain (RBG) for periodontal regeneration procedures?”

### 2.2. Eligibility Criteria

In the present systematic review, the criteria used to select the clinical studies were based on the PICOS method as follows:

- (P) Population: Adult patients with stage III-IV periodontitis presenting with residual pockets and intrabony defects with at least 3 mm of intrabony components after the completion of steps I and II of periodontal therapy (causal-related therapy; supra- and sub-gingival instrumentation) [9].
- (I) Intervention: Periodontal regeneration via the use of orally derived stem cells.
- (C) Comparison: All other strategies for periodontal regeneration.
- (O) Outcome measures:

Primary outcomes: CAL gain and PPD reduction.

Secondary outcomes: GR and RBG.

- (S) Types of studies: Only randomized controlled clinical trials (RCTs) were considered.

The following additional inclusion criteria were applied:

- Written in English language;
- At least 6 months of follow-up.

These exclusion criteria were also applied to the selection process:

- Lack of pre-treatment and post-treatment outcome measures
- Case reports, case series, retrospective studies, animal studies, and in vitro studies.

### 2.3. Search Strategy

The search was conducted through the use of various sources, both electronically and manually. The electronic research included Medline (PubMed), Scopus, and CENTRAL (Cochrane Central Register of Controlled Trials) databases. All articles published until December 2023 were searched using a combination of subject headings and free-text terms was applied. The strategy using PubMed as an example reported in Table 1. A screening of the reference lists of the included studies and related reviews was also carried out to identify any additional article of relevance. Hand searching was also implemented for the following journals: Journal of Clinical Periodontology, Journal of Dental Research, Journal of Periodontology, and Journal of Periodontal Research.

### 2.4. Study Selection

The results obtained from the manual search and from the various electronic database were downloaded and imported jointly into a reference management software, and duplicates or non-English articles were automatically removed. The identified articles were checked based on the pre-defined eligibility criteria. During the initial phase, the screening of potentially suitable titles and abstracts was performed as follows: abstracts that, at the time, met the inclusion criteria or did not provide sufficient information were admitted for the subsequent review phase. Once the eligible articles were defined, they were re-evaluated after reading the full-text by applying the selection criteria again. The studies that satisfied all the inclusion criteria were included in the systematic review. Two reviewers (A.C. and M.P.) evaluated the abstracts, titles, and full texts for selection, and, when differences occurred, they were solved via a discussion with a third party (G.O.).

### 2.5. Data Extraction for Analysis

The relevant data identified in the included studies were reported in a standardized extraction form, including the following:

- Author(s) and year of publication;
- Number of patients included in the study;
- Number of defects treated in both the test and control groups;
- Type of stem cells used in the test group;
- Type of bone defect treated;
- Type of treatment of the test group;
- Type of treatment of the control group;
- CAL gain;
- PPD reduction;
- GR;
- RBG;
- Study duration.

### 2.6. Risk of Bias of Individual Studies

The quality evaluation of the selected studies was independently performed by two review authors (A.C. and M.P.) through a risk of bias analysis, as this could impact on the overall results and conclusions. The Cochrane Collaboration’s tool was used for assessing the risk of bias [49,50]. We considered seven domains (sequence generation, allocation concealment, blinding of the outcome assessor, blinding of participants and personnel, incomplete outcome data, selective outcome reporting, and other bias) and included the results of the assessment in a specific table. Then, the overall risk of bias in the included studies was categorized as follows:

A: Low risk of bias: little chance that bias would significantly affect the outcomes if all criteria were fulfilled;

B: Unclear risk of bias: possibility of bias that could cast some doubt on the outcomes if one or more criteria were only partially met;

C: High risk of bias: likelihood of bias that could substantially undermine confidence in the outcomes if one or more criteria were not met.

### 2.7. Statistical Analysis

Studies were firstly summarized in a narrative form with key characteristics and according to the type of regenerative surgery. A meta-analysis was carried out in the presence of at least two studies of similar design. The variables were registered at the patient level. In each patient, only one tooth per technique was assessed. Weighted mean differences (MDs) and 95% confidence intervals (95% CIs) were calculated for CAL gain, PPD reduction, GR reduction [51], and RBG, using the generic inverse variance method. Forest plots were graphically depicted to summarize the differences in outcomes between the groups, using the patient as the analysis unit.

We used the χ^2^ test to assess the statistical heterogeneity among the different studies, and the percentage of variation in the global estimate due to heterogeneity was calculated using the I^2^ index (25%: low; 50%: moderate; 75% high) [49]. In case of values that are higher than 50%, the random effect method was applied. Results were considered statistically significant for *p* values < 0.05. Statistical analyses were carried out using the RevMan software version 5.4 (Copenhagen: The Nordic Cochrane Centre, The Cochrane Collaboration, 2014).

## 3. Results

### 3.1. Study Selection

**Figure 1:**
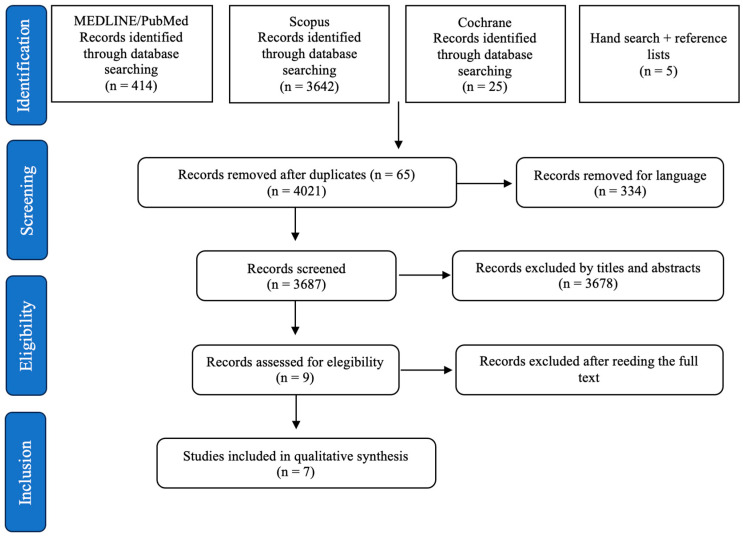
PRISMA flowchart illustrating the experimental study search and selection process.

The selection process was conducted according to the PRISMA guideline (Figure 1). The search on the MEDLINE/PubMed, Scopus, and Cochrane databases provided a total of 4086 studies; there were 65 duplicates, while 334 articles were discarded for non-English language. A number of 3687 studies were screened and, of these, 3678 were excluded after the first-stage reading of titles and abstracts due to the type of publication (chapter of book or thesis), objective, and/or design of the study. Two articles were removed after the full-text reading. Finally, seven articles met all of the inclusion criteria and were included in the qualitative analysis.

### 3.2. Risk of Bias

**Figure 2:**
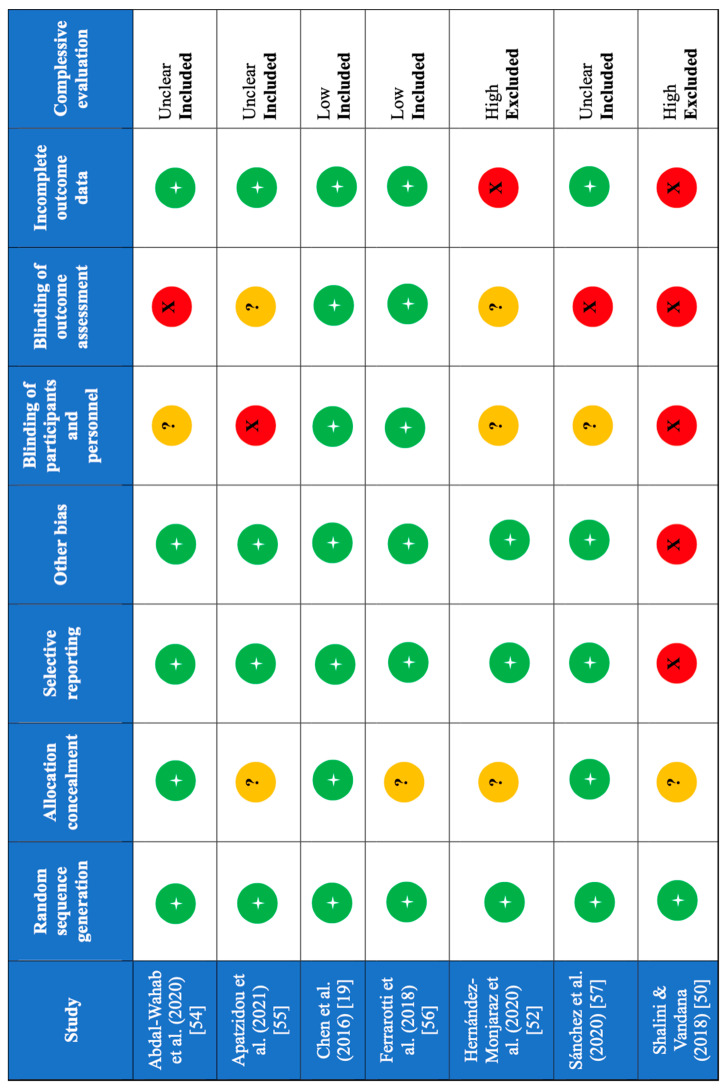
Assessment of the risk of bias in the included studies. +, criterion met; ?, unclear; X, criterion not met.

Out of the seven included RCTs, two were not included in the meta-analysis because they were rated at being of a high risk of bias [52,53]. Of the remaining five studies, three were considered as having an unclear risk of bias, and only two had a low risk of bias (Figure 2) [19,54,55,56,57]. The lack of the blinding of the outcome assessor among the seven domains was the most frequent source of bias.

### 3.3. Study Characteristics

Data extracted from the RCTs included in the review are presented in Table 2. There was a certain heterogeneity of the specific type of stem cells used in the control groups between the five studies included in the meta-analysis. Indeed, two studies [19,57] used periodontal ligament stem cells (PDLSCs), one study [56] applied dental pulp stem cells (DPSCs), one study [55] used bone marrow mesenchymal stem cells extracted from alveolar bone (ABMMSCs), and one study [54] used gingival mesenchymal stem cells (GMSCs). The follow-up lasted 6 months in one study [54] and 12 months in four studies [19,55,56,57].

Abdal-Wahab and colleagues [54] included a total of 20 patients, excluding current smokers. A full-mouth non-surgical periodontal therapy was performed in all selected patients, and they were then randomly assigned in the following test or control groups:

- Test: ten intrabony periodontal defects were treated with GMSCs associated with a β-TCP scaffold and a collagen membrane.
- Control: ten intrabony periodontal defects were treated with β-TCP and collagen membrane alone.

Apatzidou and colleagues [55] included twenty-seven patients, who were allocated into the following three groups:

- Test: nine intrabony defects were treated with ABMMSCs embedded on a collagen scaffold, enriched with a fibrin lysate and autologous platelets, using the minimally invasive surgical technique (MIST) [9].
- Control B: ten intrabony defects were treated using MIST, with only the collagen scaffold enriched with fibrin lysate and autologous platelets.
- Control C: eight intrabony defects were treated with the MIST technique alone.

Chen and colleagues [19] selected thirty patients, who were randomly assigned to one of the two groups:

- Test: 20 intrabony defects were treated with heterologous bone graft and the adjunctive use of PDLSCs.
- Control: 21 intrabony defects were treated with heterologous bone grafts only.

Ferrarotti and colleagues [56] enrolled 29 patients with severe periodontitis, randomly assigning them to one of two groups:

- Test: 15 intrabony defects were accessed with the MIST technique and were treated with DPSCs soaked onto a collagen sponge.
- Control: 14 intrabony defects were treated with only the insertion of a collagen sponge using the MIST technique.

Sánchez and colleagues [57] included a total of 20 patients. After initial periodontal therapy, the subjects were placed into one of two groups with a quasi-randomized approach, i.e., the patients assigned to the treatment group have previously undergone successful in vitro stem cell expansion processes:

- Test: 10 intrabony defects were treated with PDLSCs together with a heterologous bone substitute.
- Control: 10 intrabony defects were treated with a heterologous bone substitute alone.

### 3.4. Results of the Analyses

**Figure 3:**
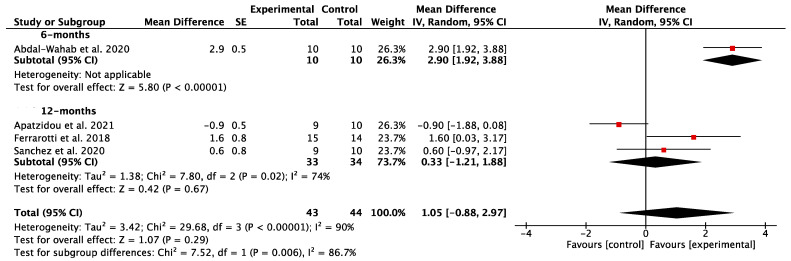
Comparison between the results of studies comparing periodontal regeneration with or without the adjunctive use of orally derived stem cells in terms of clinical attachment level (CAL) gain [54,55,56,57].

**Figure 4:**
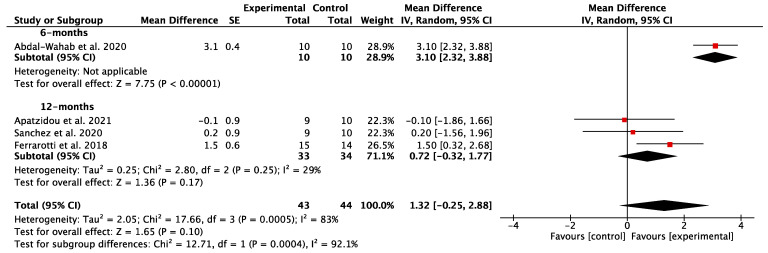
Comparison between the results of studies comparing periodontal regeneration with or without the adjunctive use of orally derived stem cells in terms of probing pocket depth (PPD) reduction [54,55,56,57].

**Figure 5:**
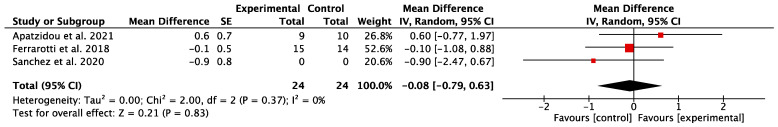
Comparison between the results of studies comparing periodontal regeneration with or without the adjunctive use of orally derived stem cells in terms of gingival recession (GR) [55,56,57].

**Figure 6:**
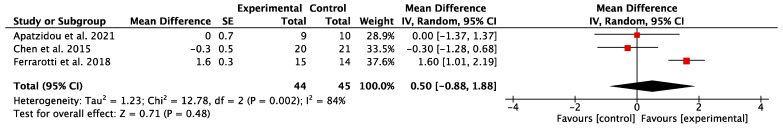
Comparison between the results of studies comparing periodontal regeneration with or without the adjunctive use of orally derived stem cells in terms of radiographical bone gain (RBG) [55,56,57].

The results of the individual studies as they relate with the main outcomes are reported in Table S1. The mean improvements reported in the meta-analyses for the different study outcomes can be summarized as follows:

- CAL gain: A total of four studies [54,55,56,57] compared the post-operative CAL gain, with a minimum of a 6-month follow-up between the test and control groups. Very high heterogeneity was encountered between the groups (*p* < 0.001; I^2^ = 90%). The meta-analysis conducted using a random-effect model revealed a non-statistically significant improvement in the test group [MD = 1.05; 95% CI (−0.88, 2.97) *p* = 0.29] (Figure 3).
- PPD reduction: A total of four studies [54,55,56,57] compared post-operative PPD reductions, with a minimum of a 6-month follow-up between the experimental group and the control group. There was a high heterogeneity between the groups (*p* < 0.001; I^2^ = 83%). A non-statistically significant adjunctive improvement in PPD reduction in the experimental group [MD = 1.32; 95% CI (−0.25, 2.88) *p* = 0.10] was shown via the meta-analysis (Figure 4).
- GR: three studies [55,56,57] compared GR between the test and the control arm with a 12-month follow-up. The meta-analysis results displayed low heterogeneity between the groups (*p* = 0.37; I^2^ = 0%), so, using a random effect model, they revealed a non-statistically significant difference between the test and control groups [MD = −0.08; 95% CI (−0.79, 0.63) *p* = 0.83] (Figure 5).
- RBG: A total of three studies [19,55,56] compared RBG between the test group and the control group. The heterogeneity was high between the groups (*p* < 0.01; I^2^ = 84%). There was no statistically significant difference in RBG between the test and control groups [MD = 0.50; 95% CI (−0.88, 1.88) *p* = 0.48] (Figure 6).

## 4. Discussion

The objective of this systematic literature review was to assess the clinical and therapeutic effectiveness of regenerative periodontal treatment when orally derived stem cells are used as adjunctive therapy through the use of RCTs. To date, MSCs can be isolated from diverse sources in the oral cavity, with dento-periodontal derived stem cells seeming to be the best candidates for periodontal tissue regeneration [37,39,58]. Therefore, the focus question of this systematic review was as follows: “In patients with periodontitis, can the adjunctive use of orally derived MSCs provide additional clinical benefits measured as CAL gain, PPD reduction, GR, and RBG for periodontal regeneration?”. A total of seven RCTs, including a total of 186 patients, were included. The risk of bias assessment led to the exclusion of two RTCs for the meta-analyses. The overall findings showed a lack of significant benefits in the adjunctive use of orally derived MSCs at 12 months during periodontal regeneration procedures. The heterogeneity in the methodology, study design, and outcomes was high.

When considering the primary outcome CAL gain, four studies were selected. The confidence interval of the data relating to the study by Apatzidou et al. [55] and the study by Sánchez et al. [57] exceeded the vertical line of the reference value, meaning there was no statistically significant difference between the test and control groups. Conversely, Ferrarotti et al. [56] and Abdal-Wahab et al. [54] provided a statistically significant advantage for the adjunctive application of MSCs both at the 6- and 12-month follow-up. When the studies were combined, the final meta-analysis revealed a non-statistically significant improvement of 1.05 mm in CAL gain for the test group [95% CI (−0.88, 2.97) *p* = 0.29]. For PPD reduction, the same four studies were selected [54,55,56,57], with the overall result of the meta-analysis being not statistically significant at 12 months, but with a significant advantage in the study at 6 months [54]. This finding may suggest a greater rapidity of the periodontal regeneration process following the use of MSCs, although, in the long term, the results of the regenerative surgical treatment appear to be comparable to those of other regenerative methods. When delving deeper into study characteristics, Ferrarotti et al. [56] showed the highest difference in outcome measures between the test and control group with respect to other included studies with a low risk of bias. This discrepancy can be both ascribed to (i) the use of DPSCs and (ii) the nature of the regenerative procedure in the control group. Indeed, DPSCs hold significant promise due to their accessibility, shared origin, and similar antigenic pattern to PDLSCs, making them particularly attractive for therapeutic applications [33]. DPSCs exhibit an extended lifespan, display compatibility with biomaterials, and can be safely preserved through cryopreservation methods [59]. Building on this foundation, experimental findings from studies conducted in vivo and in animal models suggest that DPSCs have the capability to produce lamellar bone with proper vascularization. Moreover, DPSCs demonstrate the potential for differentiation into various periodontal tissues, emphasizing their versatility and potential therapeutic efficacy in the field of periodontal regeneration [59,60]. Regarding PDLSCs, they can be found both on the root and alveolar bone surfaces after tooth extraction, although those on the root demonstrate superior differentiation capabilities [61]. Recognized for their safety and efficacy, they became the pioneering treatment in periodontal regeneration therapy [35,41,62]. Indeed, PDLSCs exhibited the ability to differentiate into mesenchymal cell lineages, generating cells which were capable of forming collagen, adipocytes, cementum tissue, Sharpey’s fibers, and osteoblast-like cells in vivo [63]. However, the translatability of both DPSCs and PDLSCs to the clinics has been hindered by several limitations, including the necessity for tooth extraction and the possibility that chronic exposure to a chronic inflammatory environment could lead to the depletion of their potential through senescence [36]. Finally, bone marrow-derived mesenchymal stem cells are a specific type of multipotent MSCs that can be obtained from the alveolar bone during surgery, providing comparable biologic features to iliac BMMSCs [64]. They have also shown the potential of inducing not only the reconstruction of bone, but also periodontal and dental tissue regeneration in preclinical models. Indeed, they have the ability to increase the expression of genes related to tooth development, and they can transform into cells resembling ameloblasts and periodontal tissue cells [65]. Lastly, although presenting a biological rationale to hypothesize their use [66], no study testing the application of adipose-derived stem cells in periodontal tissue regeneration was found.

Three recent systematic reviews focusing on this topic are present in the literature [46,47,67], with their results and conclusions disagreeing substantially from the present study. Indeed, their meta-analyses revealed significant differences between the experimental and control groups in terms of PPD, CAL, radiographic intrabony defect depth, and GR, emphasizing how the use of MSCs can be beneficial in periodontal regeneration. In contrast to these optimistic trends, the present systematic review revealed an overall lack of significant benefits after 12 months. Notably, the observed heterogeneity in the methodology, study design, and measured outcomes was consistently pronounced. Indeed, in a plausible attempt to broaden the focus, a previous systematic review combined studies using MSCs derived from diverse body sources (such as umbilical stem cells) for different oral surgical interventions (i.e., alveolar bone reconstruction) at different time-points (3, 6, and 12 months). Indeed, the inclusion of multiple follow-up groups from the same RCT may lead to excessive weight in the meta-analyses. Overall conclusions cannot overlook these important heterogeneities, if they are to provide a clear snapshot of the state of the art or guide future research endeavors. This raises important considerations about the standardization of protocols and the need for more homogeneity in future research endeavors, elucidating the specific conditions under which orally derived MSCs may or may not be effective in enhancing periodontal regeneration. Also, it is important to underline the fact that the included studies evaluated differences in term of clinical and radiographic outcomes, without histological proof of regeneration. To circumvent ethical concerns, future studies may use pro-resolving or early healing markers in the gingival crevicular fluid in order to explore more biologically the potential advantage of using MSCs [68,69,70].

The attention towards the use of MSCs in periodontal therapy derives from the need to implement treatment options for lesions resulting from periodontitis, due to its global prevalence [71]. In recent years, MSCs have achieved increasing success in the treatment of many pathologies studied by various branches of medicine, based on their regenerative and immunoregulatory properties. Our knowledge is still limited, and this means that the prospects regarding their clinical use are very broad. It should be considered that there are critical steps to increase the translatability of stem cell-based therapeutic approaches. In fact, it must be said that the safety of cell therapies, in general, has not yet been fully evaluated. Notably, no RCT in the present review showed adverse events in terms of the use of dento-periodontal-derived stem cells. Furthermore, questions regarding cell delivery, immunogenicity, the use of autologous or allogeneic cells, culture quality control, and cost effectiveness are critical to address. The next phase of research should aim to identify the tissues that can optimally serve as the source of stem cells, and, in this sense, future attention should be even more focused on dental and periodontal tissues. It should be emphasized that, until recently, medical stem cell research has not prioritized periodontal tissues due to the non-life-threatening nature of periodontitis.

Although efforts were made to enhance the quality of data regarding the topic, this study has certain limitations, primarily stemming from the nature of the existing literature. Indeed, the RCTs provided periodontal regeneration with very diverse flap design, scaffolds, MSCs vectors, and cell-handling technologies. Despite the promising outcomes highlighted in the broader literature, our synthesis points to a nuanced perspective, suggesting that the use of orally derived MSCs may not consistently confer additional clinical benefits in the specified timeframe. While recognizing the potential of stem cell therapies, including MSCs, our findings underscore the complexity of translating these approaches into consistently successful clinical outcomes in the context of periodontitis.

## 5. Conclusions

In conclusion, it was not possible to demonstrate that the additional use of dento/periodontal stem cells in periodontal regenerative surgical procedures determines an improvement in clinical and radiographic parameters when compared to other biomaterials or techniques which are more studied in the literature. The regenerative approach supported by tissue engineering and cell therapy should be explored in depth with a significantly higher number of randomized controlled clinical trials, with larger samples and at least 12 months of follow up to allow for the detection of long-term outcomes. In consideration of the results expressed, the low number of RCTs, the inherent costs of using MSCs, and the possibility of adverse events still under-addressed in the literature, regenerative periodontal surgery with the use of stem cells for bone defects could not be currently considered as a preferential approach for clinical treatment over other periodontal regeneration procedures.

## 6. Indications for Future Research

- RCTs evaluating the clinical efficacy, as well as patient-related outcomes and cost–benefit analyses of periodontal regeneration using dento-periodontal stem cells.
- This RCTs should be designed with an increased number of patients enrolled and a long-term follow-up.
- Studies focusing on clinical protocols to obtain an efficient number of MSCs from the oral cavity.
- Studies focusing on side effects in both the short and long term via the use of MSCs.

## Figures and Tables

**Table 1:** Search strategy.

(Periodontal defect OR periodontal lesion OR periodontal osseous defect OR intraosseous defect OR intra-osseous defect OR intrabony defect OR infra-bony defect OR angular defect OR bony defect OR osseous defect OR crater)
AND
(stem cells OR stem OR stem cell therapy OR cell therapy OR MSC OR mesenchymal stem cells OR human cord stem cells OR BMMSC OR bone marrow mesenchymal stem cells OR pluripotent stem cells OR embryonic stem cells OR ESC OR cell technology OR oral stem cells OR stem cell-delivery therapeutics OR induced pluripotent stem cells OR iPSC OR adipose-derived stem cells OR dental stem cells OR pulp stem cells OR periodontal ligament stem cells OR PDLSC OR progenitor cells OR apical papilla stem cells OR dental follicle stem cells OR human exfoliated deciduous tooth cells)
AND
(clinical trial OR case report OR prospective study OR longitudinal study OR cohort study OR RCT OR randomized clinical trial)
AND
(GTR OR guided tissue regeneration OR periodontal regeneration)

**Table 2:** Summary of studies included in the systematic review.

Study	MSC Type	Defect Inclusion Criteria	Group Characteristics		Number of Patients		Number of Defects		Primary Outcomes
			Test	Control	Test	Control	Test	Control	
Apatzidou et al., 2021 [55]	Autologous alveolar bone marrow mesenchymal stem cells (ABMMSCSs)	Infrabony defect (5 1-wall defects in the control groups; 3 2-wall defects in the test group and 3 in the control groups; 6 3-wall defects in the test group and 10 in the control groups)	ABMMSCs + autologous fibrin/platelet lysate (aFPL) + collagen scaffold + MIST	Group B: autologous fibrin/platelet lysate (aFPL) + collagen scaffold + MIST Group C: MIST	9	10 + 8	9	Group B: 10 Group C: 8	CAL; PPD; GR; BDD; BC-BD
Sanchez et al., 2020 [57]	Autologous periodontal ligament-derived mesenchymal stem cells (PDLSCs)	Infrabony defect (3 1-wall defects in the test group; 7 2-wall defects in the test group and 10 in the control group)	PDLSCs + bone xenograft	Bone xenograft	10	10	10	10	CAL; PPD; GR
Abdal-Wahab et al., 2020 [54]	Autologous gingival-associated mesenchymal stem cells (GMSCs)	Infrabony defect (7 2-wall defects in the test group and 6 in the control group; 3 3-wall defects in the test group and 4 in the control group)	GMSC + (beta-tricalcium phosphate (β-TCP) + collagen membrane	Beta-tricalcium phosphate (β-TCP) + collagen membrane	10	10	10	10	CAL; PPD
Hernández-Monjaraz et al., 2020 [52]	Autologous dental pulp stem cells (DPSCs)	Infrabony defects	DPSCs + collagen scaffold	Collagen scaffold	11	10	11	10	PPD
Ferrarotti et al., 2018 [56]	Autologous dental pulp stem cells (DPSCs)	Infrabony defect (7 1-wall defects in the test group and 5 in the control group; 4 2-wall defects in the test group and 5 in the control group; 4 3-wall defects in the test group and 4 in the control group)	DPSCs + MIST + collagen sponge	MIST + collagen sponge	15	14	15	14	CAL; PPD; GR; BC-BD
Shalini and Vandana 2018 [53]	Autologous periodontal ligament-derived mesenchymal stem cells (PDLSCs)	Infrabony defects	OFD + PDLSCs	OFD	14	14	14	14	CAL, PPD
Chen et al., 2016 [19]	Autologous periodontal ligament-derived mesenchymal stem cells (PDLSCs)	Infrabony defect (Defect characteristics not mentioned)	PDLSCs + GTR + Bio-oss	GTR + Bio-oss			20	21	CAL; PPD; GR; BDD

Legend: CAL: clinical attachment level; PPD: probing pocket depth; GR: gingival recession; BDD: linear distance from cementoenamel junction to bottom of defect; BC-BD: linear distance from bone crest to bottom of defect; MIST: minimally invasive surgical technique; MSC: mesenchymal stem cell; GTR: guided tissue regeneration; OFD: open flap debridement.

## Data Availability

The original contributions presented in the study are included in the article and Supplementary Materials, further inquiries can be directed to the corresponding author.

## Supplementary Materials

The following supporting information can be downloaded at https://www.mdpi.com/article/10.3390/dj12050145/s1, Table S1: Included studies: summary of the results for the main outcomes of interest.

## Abbreviations

MD (mean difference); CAL (clinical attachment level); PPD (probing pocket depth); GR (gingival recession); RGB (radiographic bone gain); CI (confidence interval); BoP (bleeding on probing); FI (furcation involvement); MSCs (mesenchymal stem cells); RCTs (randomized clinical trials); BMMSC (bone marrow mesenchymal stem cells); ESC (embryonic stem cells); iPSC (induced pluripotent stem cells); PDLSCs (periodontal ligament stem cells); GTR (guided tissue regeneration); DPSCs (dental pulp stem cells); ABMMSCs (alveolar bone marrow mesenchymal stem cells); GMSCs (gingival mesenchymal stem cells); MIST (minimally invasive surgical technique); BDD (linear distance from cementoenamel junction to bottom of defect); BC-BD (linear distance from bone crest to bottom of defect); OFD (open flap debridement).
