# Supplementary material for: Orally Derived Stem Cell-Based Therapy in Periodontal Regeneration: A Systematic Review and Meta-Analysis of Randomized Clinical Studies
Source: Dent J (Basel). 2024 May 16;12(5):145. doi: 10.3390/dj12050145 (PMC11120617; doi:10.3390/dj12050145)
Supplement: Supplementary file 1 [file dentistry-12-00145-s001.zip › dentistry-2943245-supplementary.pdf]

Table S1. Included studies: summary of the results for the main outcomes of interest.

Study	Group	CAL			PPD			Radiographic bone levels			GR		
		**Baseline**	**6 months**	**12 months**	**Baseline**	**6 months**	**12 months**	**Baseline**	**6 months**	**12 months**	**Baseline**	**6 months**	**12 months**
Apatzidou et al. 2021 [55]	Test A	7.3 ± 1.5	5.0 ± 1.8 (Δ 2.3 ± 1.2)	4.6 ± 1.6 (Δ 2.8 ± 1.0)	6.7 ± 1.6	3.9 ± 1.5 (Δ 2.8 ± 0.8)	3.2 ± 1.2 (Δ 3.4 ± 1.1)	BDD: 7.2 ± 2.8<br>BC-BD: 4.9 ± 2.2	NA	5.1 ± 2.8<br>2.8 ± 2.2	0.7 ± 1.0	1.1 ± 1.6	1.3 ± 1.7
	Control B	9.1 ± 2.7	6.5 ± 1.5 (Δ 2.6 ± 2.3)	6.0 ± 1.0 (Δ 3.1 ± 2.4)	7.6 ± 2.42	4.5 ± 1.2 (Δ 3.1 ± 2.5)	3.4 ± 1.0 (Δ 3.5 ± 2.3)	BDD: 8.2 ± 2.3<br>BC-BD: 4.3 ± 0.9	NA	7.7 ± 2.2<br>2.8 ± 0.7	1.5 ± 1.3	2.0 ± 1.2	1.9 ± 1.4
	Control C	8.9 ± 2.7	5.6 ± 1.7 (Δ 3.3 ± 1.5)	5.8 ± 1.6 (Δ 3.1 ± 2.0)	7.5 ± 1.8	3.6 ± 1.1 (Δ 3.9 ± 1.0)	3.3 ± 1.2 (Δ 4.2 ± 1.9)	BDD: 8.3 ± 2.9<br>BC-BD: 5.9 ± 2.8	NA	6.8 ± 1.9<br>3.9 ± 1.9	1.4 ± 1.2	2.0 ± 0.8	2.5 ± 1.1
Sanchez et al. 2020 [57]	Test	10.6 ± 2.2	NA	9.44 ± 2.3 (Δ 1.44 ± 1.87)	6.6 ± 1.07	NA	4.3 ± 2.1 (Δ 2.3 ± 1.3)	NA	NA	NA	4.0 ± 1.7	NA	5.3 ± 2.2
	Control	9.9 ± 1.6	NA	9.1 ± 2.1 (Δ 0.8 ± 1.68)	6.8 ± 1.03	NA	4.7 ± 2.1 (Δ 2.1 ± 2.46)	NA	NA	NA	3.2 ± 1.8	NA	4.4 ± 1.3
Abdal-Wahab et al. 2020 [54]	Test	6.3 ± 2.06	2.3 ± 1.16	NA	7.5 ± 2.42	3.10 ± 0.88	NA	NA	NA	NA	NA	NA	NA
	Control	5.3 ± 0.95	4.20 ± 1.0	NA	6.5 ± 0.52	5.20 ± 0.80	NA	NA	NA	NA	NA	NA	NA
Hernández-Monjaraz et al. 2020 [52]	Test	NA	NA	NA	5.66 ± 0.41	2.34 ± 0.29 (Δ 3.32 ± 0.12)	NA	NA	NA	NA	NA	NA	NA
	Control	NA	NA	NA	5.58 ± 0.38	3.78 ± 0.53 (Δ 1.80 ± 0.15	NA	NA	NA	NA	NA	NA	NA
Ferrarotti et al. 2018 [56]	Test	10.0 ± 1.6	5.4 ± 1.2 (Δ 4.6 ± 1.4)	5.5 ± 1.1 (4.5 ± 1.9)	8.3 ± 1.2	3.5 ± 0.8 (Δ 4.8 ± 0.9)	3.4 ± 0.9 (Δ 4.9 ± 1.4)	BC-BD: 6.4 ± 1.4	2.7 ± 0.8	2.5 ± 0.7	1.7 ± 1.2	1.9 ± 1.2	2.1 ± 1.3
	Control	9.4 ± 1.5	6.6 ± 1.3 (Δ 2.8 ± 1.7)	6.5 ± 1.2 (Δ 2.9 ± 2.2)	7.9 ± 1.3	4.6 ± 1.0 (Δ 3.3 ± 1.6)	4.5 ± 1.0 (Δ 3.4 ± 1.7)	BC-BD: 5.6 ± 1.0	4.1 ± 0.9	4.1 ± 0.9	1.5 ± 0.8	2.0 ± 1.1	2.0 ± 1.2
Shalini & Vandana 2018 [53]	Test	6.35 ± 1.15	NA	NA	8.28 ± 1.26	NA	NA	NA	NA	NA	NA	NA	NA

	Group	6.92 ± 2.55	NA	NA	8.21 ± 3.06	NA	NA	NA	NA	NA	NA	NA	NA
Chen et al. 2016 [19]	Test	NA	NA	NA	NA	NA	NA	BDD: 7.20 ± 2.65	4.61 ± 1.87	4.49 ± 2.03	NA	NA	NA
	Control	NA	NA	NA	NA	NA	NA	BDD: 7.19 ± 1.87	5.11 ± 1.53	4.80 ± 1.41	NA	NA	NA

Legend: CAL: clinical attachment level; PPD: probing pocket depth; BDD: linear distance from cementoenamel junction to bottom of defect; BC-BD: linear distance from bone crest to bottom of defect.
